# On metabolic reprogramming and tumor biology: A comprehensive survey of metabolism in breast cancer

**DOI:** 10.18632/oncotarget.11759

**Published:** 2016-08-31

**Authors:** Judith Penkert, Tim Ripperger, Maximilian Schieck, Brigitte Schlegelberger, Doris Steinemann, Thomas Illig

**Affiliations:** ^1^ Institute of Human Genetics, Hannover Medical School, Hannover, Germany; ^2^ Hannover Unified Biobank, Hannover Medical School, Hannover, Germany

**Keywords:** cancer metabolism, breast cancer, metabolomics, cancer microenvironment, tumor heterogeneity

## Abstract

Altered metabolism in tumor cells has been a focus of cancer research for as long as a century but has remained controversial and vague due to an inhomogeneous overall picture. Accumulating genomic, metabolomic, and lastly panomic data as well as bioenergetics studies of the past few years enable a more comprehensive, systems-biologic approach promoting deeper insight into tumor biology and challenging hitherto existing models of cancer bioenergetics. Presenting a compendium on breast cancer-specific metabolome analyses performed thus far, we review and compile currently known aspects of breast cancer biology into a comprehensive network, elucidating previously dissonant issues of cancer metabolism. As such, some of the aspects critically discussed in this review include the dynamic interplay or metabolic coupling between cancer (stem) cells and cancer-associated fibroblasts, the intratumoral and intertumoral heterogeneity and plasticity of cancer cell metabolism, the existence of distinct metabolic tumor compartments in need of separate yet simultaneous therapeutic targeting, the reliance of cancer cells on oxidative metabolism and mitochondrial power, and the role of pro-inflammatory, pro-tumorigenic stromal conditioning. Comprising complex breast cancer signaling networks as well as combined metabolomic and genomic data, we address metabolic consequences of mutations in tumor suppressor genes and evaluate their contribution to breast cancer predisposition in a germline setting, reasoning for distinct personalized preventive and therapeutic measures. The review closes with a discussion on central root mechanisms of tumor cell metabolism and rate-limiting steps thereof, introducing essential strategies for therapeutic targeting.

For a tumor to arise, it has long been common understanding that a critical degree of accumulating factors jointly contribute to clonal evolution. These include but are not limited to (i) a proliferation advantage mediated in part by the activation of proto-oncogenes (e.g. *MYC, RAS*, and/or PI3K-AKT-mTOR pathway components) and (ii) the disturbance of cellular control mechanisms due to mutations in tumor suppressor genes involved in cell cycle control (*TP53, CHEK2*), DNA repair (*BRCA1, BRCA2*), or proliferation-restrictive signaling (*PTEN*). When present at germline level, these variations constitute a potential shortcut in clonal evolution and predispose affected individuals to tumor development. However, impaired DNA repair mechanisms, aberrant cell cycle control, and genomic instability alone may not be sufficient for malignant transformation as rapid proliferation can only be possible in the presence of sufficient macromolecule supply for building blocks and ATP for energy. Accumulative evidence suggests that, in order for tumors to emerge and particularly to evolve into a more aggressive state, a third component (iii), intertwined with (i) and (ii), may be mandatory - the metabolic reprogramming of tumor cells including their surrounding stromal environment. This metabolic switch has been entitled one of the new “hallmarks of cancer” [1], expanding the original set of hallmarks [2] along with further upgrades such as chronic inflammation, escape from the immune system, and genomic instability. Metabolome analyses on tumor tissue and cancer patients' biofluids aim for a better understanding of metabolic reprogramming and malignant transformation in tumor cells, and integration of the underlying mechanisms and signaling pathways is expected to help elucidate overall tumor pathogenesis as well as breast cancer pathogenesis in particular, the latter constituting the main focus of the present review.

## METABOLIC TUMOR MODELS AND TUMOR BIOLOGY

**Figure 1:**
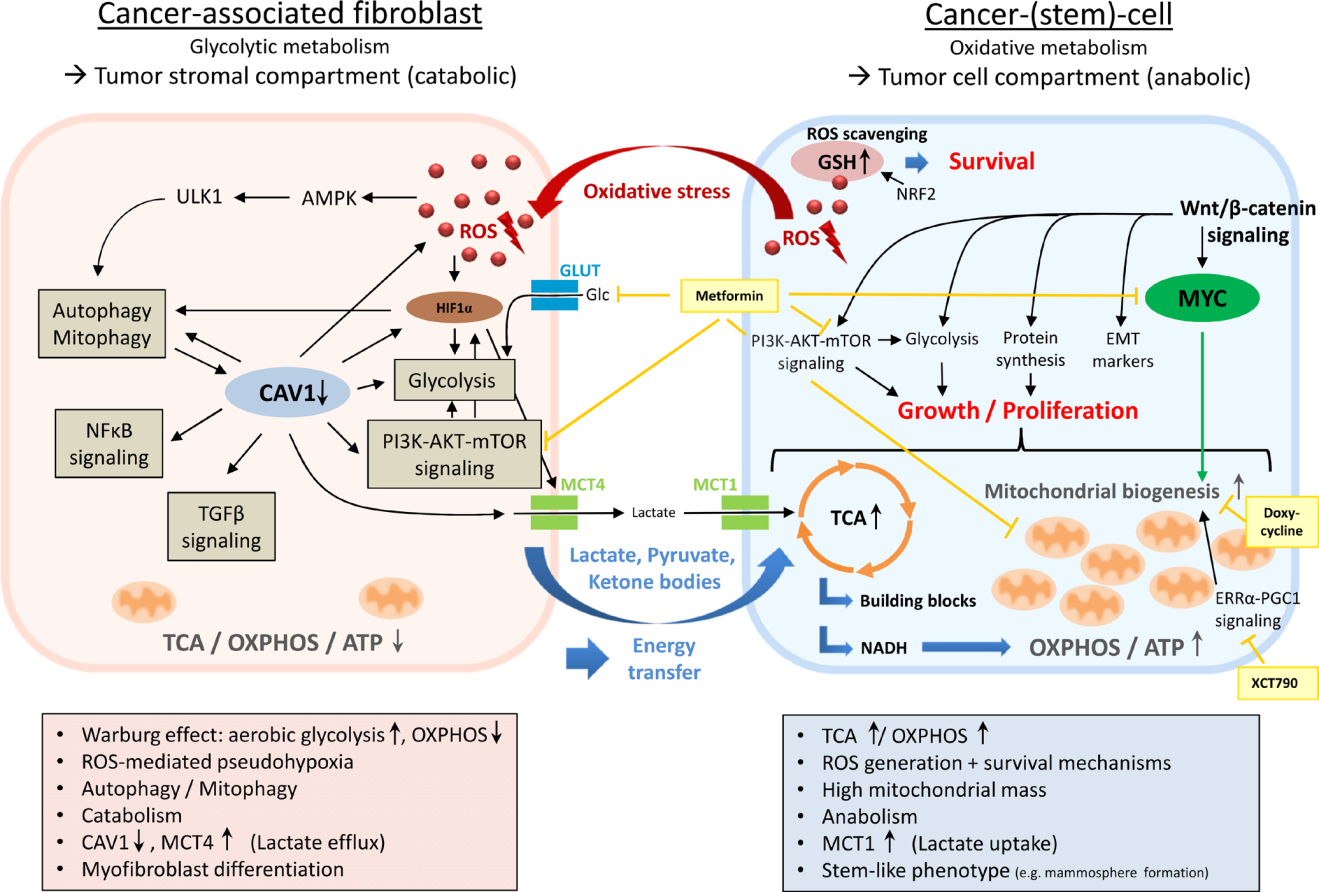
Metabolic coupling in two-compartment tumor metabolism In this model of metabolic symbiosis, the dynamic interplay between distinct tumor compartments (i.e. the tumor stromal compartment and the tumor cell compartment) enables cancer cells to acquire anapleurotic substrates for energy production and proliferation through paracrine supply of glycolytic end products derived from cancer-associated fibroblasts (CAFs). Cancer cells mediate metabolic reprogramming of fibroblasts into CAFs *via* the secretion of ROS, creating a pseudohypoxic state in adjacent stroma cells. Due to the simultaneous upregulation of innate antioxidant pathways (e.g. NRF2 signaling, GSH replenishment), cancer cells protect themselves from excessive oxidative damage. In the tumor stromal compartment, ROS accumulation initiates fibroblast-to-myofibroblast differentiation and transformation into a pro-inflammatory, catabolic phenotype producing high amounts of macromolecules (e.g. lactate, pyruvate, ketone bodies) due to a major bioenergetic emphasis on aerobic glycolysis (Warburg effect). The ROS-triggered loss of CAV1 plays a vital role in this process by promoting the upregulation of HIF1α, NFκB, TGFβ, glycolysis, autophagy/mitophagy, and oxidative stress in a feed-forward cycle manner. Loss of CAV1 is associated with high MCT4 expression - a marker of lactate efflux and aerobic glycolysis. Anapleurotic substrates derived from CAFs' excessive glycolytic activity are subsequently transferred into adjacent cancer cells where they induce TCA/OXPHOS, enabling the generation of ample amounts of ATP as well as macronutrients for proliferation. High mitochondrial mass, a new feature of the stem-like phenotype associated with upregulated Wnt/β-catenin-, MYC-, and ERRα-PGC1-signaling, reflects cancer cells' reliance on oxidative metabolism. AMPK, AMP-activated protein kinase; CAV1, caveolin 1; EMT, epithelial-mesenchymal transition; ERRα, estrogen-related receptor alpha; Glc, glucose; GLUT, glucose transporter; GSH, glutathione (reduced form); HIF1α, hypoxia-inducible factor 1-alpha; MCT1, monocarboxylate transporter 1; MCT4, monocarboxylate transporter 4; NRF2, nuclear factor E2-related factor 2; OXPHOS, oxidative phosphorylation; PGC1, peroxisome proliferator-activated receptor γ coactivator 1; ROS, reactive oxygen species; TCA, tricarboxylic acid cycle; ULK1, unc-51-like kinase 1; Light yellow boxes, medical interventions.

Originally described by Otto Warburg [3], metabolic reprogramming in cancer cells was thought to involve a shift in energy metabolism away from an oxidative towards a glycolytic one - even under aerobic conditions - subsequently termed the “Warburg effect” or “aerobic glycolysis”. Dysfunctional mitochondrial oxidative phosphorylation (OXPHOS) was proposed to play a major causative role in this process. However, in recent years this concept of mitochondrial respiratory impairment has been challenged and a growing body of evidence now suggests that, indeed, high mitochondrial activity and even a dependence on mitochondrial metabolism - as well as glycolysis - is essential for rapid tumor cell proliferation. This principle (reviewed in [4]) is supported by the observation that depletion of mitochondrial DNA (mtDNA) halts cancer cell proliferation and reverses tumorigenicity. The original Warburg effect *can* instead be found in cells belonging to the surrounding stromal tumor microenvironment, e.g. cancer-associated fibroblasts (CAFs), which are believed to stand in a quasi-symbiotic relationship with cancer cells (Figure 1). In an autophagy-associated paracrine manner, CAFs supply adjacent cancer cells with anapleurotic substrates such as lactate, pyruvate, and ketone bodies derived from their own excessive glycolytic activity. Hereby, they induce OXPHOS in adjacent cancer cells, enabling them to produce ample amounts of ATP as well as supplying necessary macronutrients for proliferation. This model of two-compartment tumor metabolism and the dynamic interplay or “near-parasitic” metabolic symbiosis between cancer cells and CAFs has been redefined as the “reverse Warburg effect” [5] or “metabolic coupling”. Apart from providing a foundation for better interpretation of tumor-associated metabolome analyses, this concept may have broad implications for (i) the elucidation of fundamental aspects of tumor etiology and pathogenesis, (ii) the enhancement of diagnostic accuracy and classification, and (iii), above all, the development of preventive and therapeutic approaches, focusing in parallel on (a) basic metabolic root tumor mechanisms as well as (b) precise personalized treatment according to individual metabolic tumor-specific aberrations.

**Figure 2:**
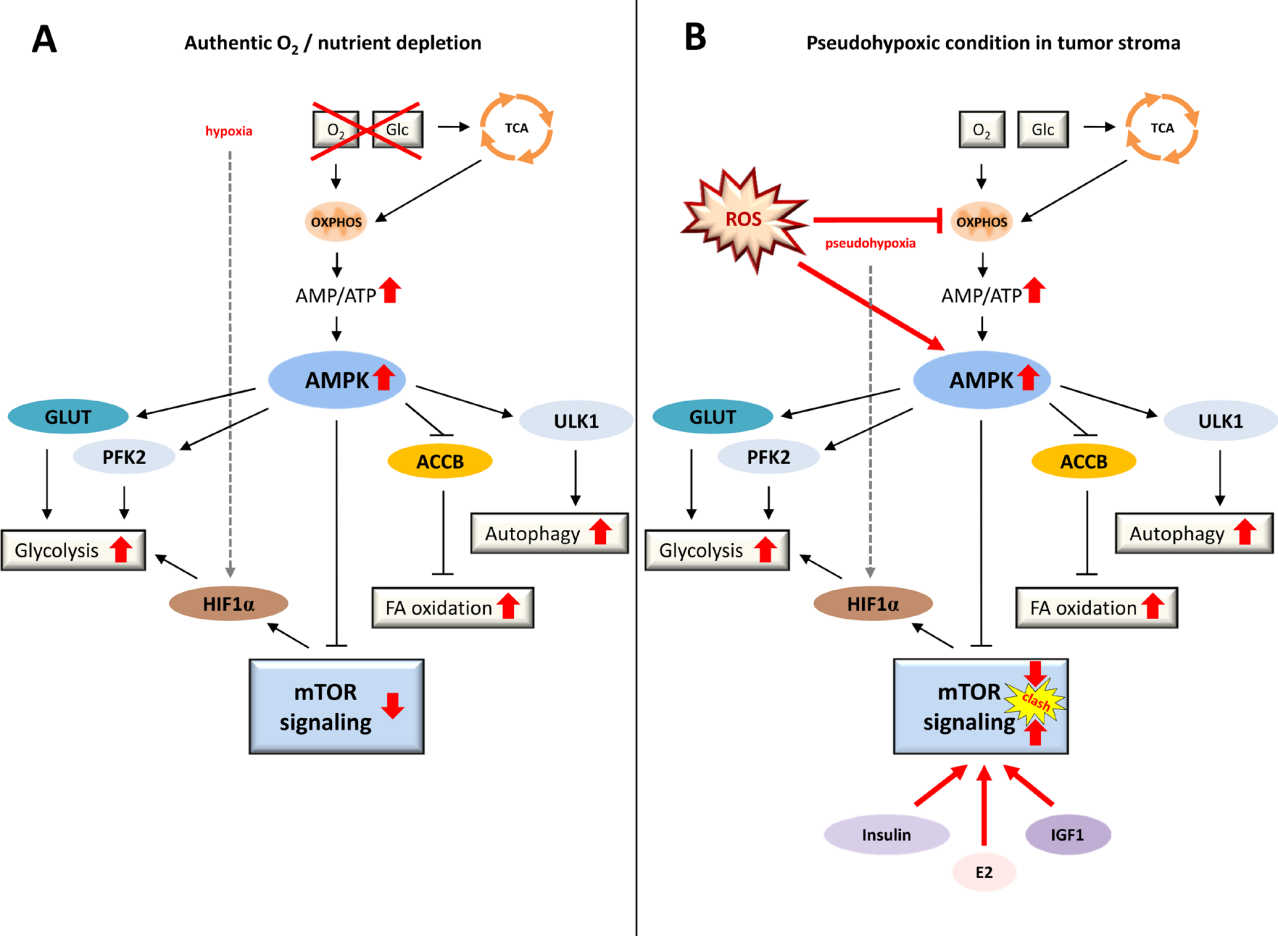
Conditions of authentic energy starvation *versus* tumor-associated pseudohypoxia **A.** Under physiological conditions, a state of energy starvation leads to a lack of fuel for OXPHOS resulting in lower ATP output, increased AMP/ATP ratios, and activated AMPK, ultimately promoting glycolysis, fatty acid oxidation, autophagy and anti-proliferative signaling *via* mTORC1 inhibition. These signaling reactions are of major advantage to a cell or an organism under conditions of starvation. **B.** In a tumor setting, in contrast, cancer cells mimic a state of energy depletion in the tumor stromal compartment, even though nutrient supply is ample and growth factors constantly trigger pro-proliferative PI3K-AKT-mTOR signaling. Thus, in a state of authentic nutrient and oxygen depletion (A) mTOR signaling is physiologically downregulated by AMPK, whereas in a state of pseudohypoxia provoked in fibroblasts by surrounding cancer cells (B) autophagy-promoting AMPK signaling clashes with elevated PI3K-AKT-mTOR signaling - a condition that leads to a major excess of macromolecules. ACCB, acetyl-CoA-carboxylase-beta; AMP, adenosine monophosphate; AMPK, AMP-activated protein kinase; E2, estradiol; FA oxidation, fatty acid oxidation; Glc, glucose; GLUT, glucose transporter; HIF1α, hypoxia-inducible factor 1-alpha; IGF1, insulin-like growth factor 1; mTOR, mechanistic target of rapamycin; OXPHOS, oxidative phosphorylation; PFK2, phosphofructokinase 2; ROS, reactive oxygen species; TCA, tricarboxylic acid cycle; ULK1, unc-51-like kinase 1.

Mechanistically, metabolic reprogramming (Figure 1) of fibroblasts into a CAF phenotype is mediated by cancer cells through the generation and secretion of high amounts of hydrogen peroxide (H_2_O_2_), which creates a pseudohypoxic state in adjacent stroma cells, mimicking nutrient and oxygen depletion [6]. Cancer cells are protected from excessive damage by reactive oxygen species (ROS) through the simultaneous upregulation of innate protective antioxidant pathways *via* mechanisms such as nuclear factor, erythroid 2 like 2 (NFE2L2) stabilization (also known as NRF2) and generation of reduced glutathione (GSH) [7, 8]. Physiologically, a state of energy starvation - in particular a shortage of glucose or oxygen - leads to the following signaling scenario (Figure 2A). The lack of fuel for OXPHOS results in lower ATP output and increased AMP/ATP ratios, subsequently activating AMP-activated protein kinase (AMPK) and resulting in (i) the activation of glycolysis through (a) phosphorylation of phosphofructokinase 2 (PFK2), and (b) elevated glucose uptake by means of glucose transporter type 4 (*GLUT4*) expression, (ii) the activation of fatty acid oxidation through inhibition of acetyl-CoA-carboxylase-beta (ACCB), (iii) the induction of anti-proliferative signaling through an enhancement of AMPK's inhibitory effect on mechanistic target of rapamycin (mTOR), and (iv) the onset of an autophagic, self-digestive condition through direct phosphorylation of unc-51-like kinase 1 (ULK1) in order to recycle any nonessential cell components for nutrients and for the highest possible energy exploitation [9] (http://www.kegg.jp/kegg-bin/highlight_pathway?scale=1.0&map=map04152&keyword=sirt1; 02/05/2016). Conceivably, all of these signaling reactions are of major advantage to a cell or an entire organism under conditions of starvation. In a tumor setting (Figure 2B), however, cancer cells divert this mechanism towards their own advantage by mimicking a state of energy depletion in surrounding cells even though nutrient supply is ample and constantly triggers the growth-promoting, pro-proliferative PI3K-AKT-mTOR pathway *via* growth factors such as insulin and insulin-like growth factor 1 (IGF1). Whereas in a state of authentic nutrient and oxygen depletion (Figure 2A) mTOR signaling is physiologically downregulated by AMPK, the ROS-mediated pseudohypoxic state cancer cells provoke in surrounding fibroblasts (Figure 2B) results in simultaneous and persistent activation of autophagy-promoting AMPK signaling, on the one hand, and elevated mTOR signaling, on the other hand - an unphysiological condition, leading to macromolecule abundance and catabolism.

**Figure 3:**
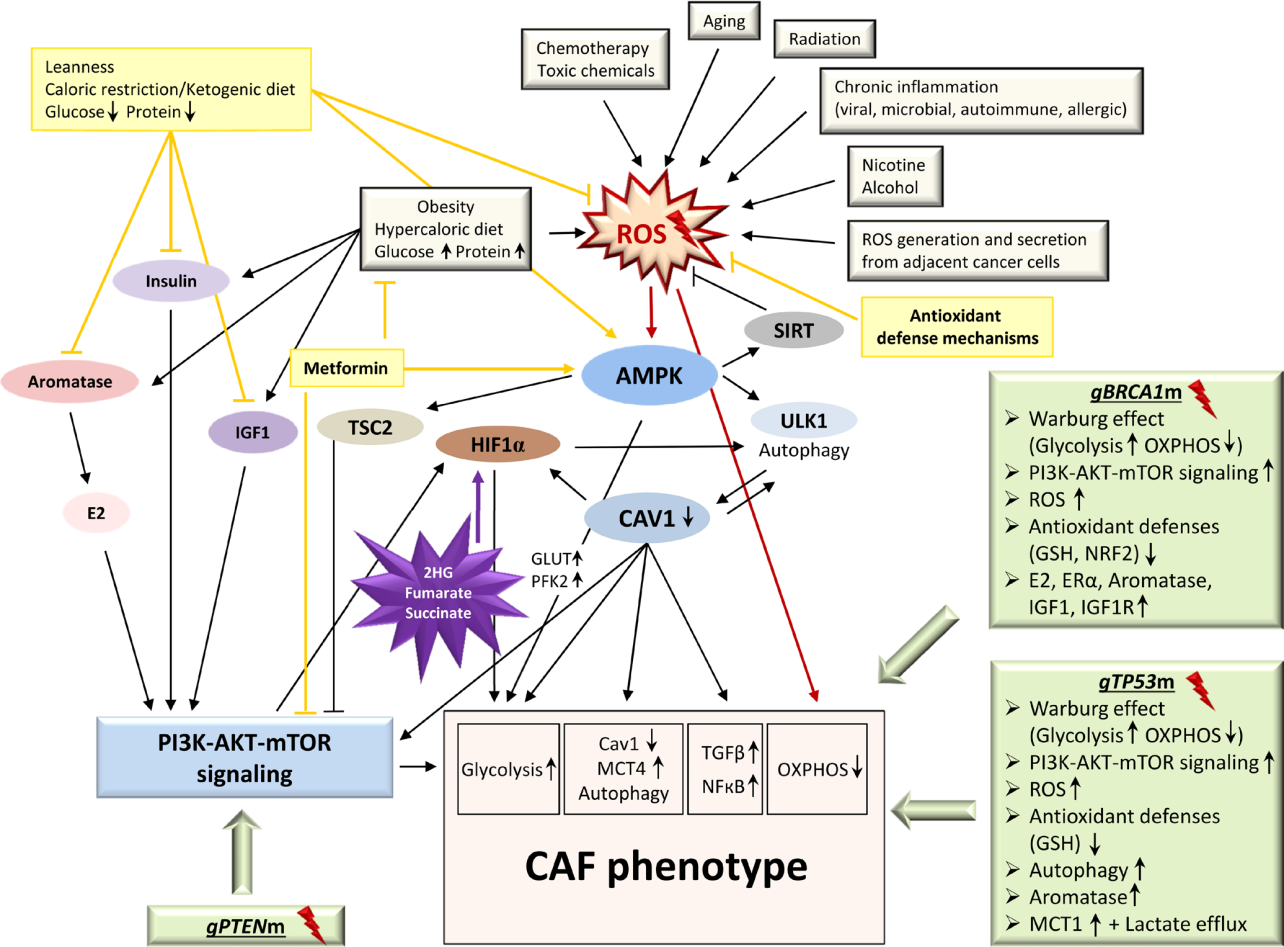
The CAF phenotype: onset and triggers The onset of a pro-inflammatory, pro-tumorigenic stromal environment by metabolic reprogramming of fibroblasts into a CAF phenotype can be triggered by a variety of factors including genetic (light green boxes) and environmental (light grey boxes) aspects. The growing burden of accumulating factors likely assists cancer-(stem)-cells in creating a catabolic, glycolytic, and autophagic stromal environment to their needs. Consequently, any type of prolonged ROS accumulation, overactive PI3K-AKT-mTOR signaling, or favoring of glycolytic flux over OXPHOS may help set the conditions for a metabolic switch. This likely includes germline aberrations in tumor suppressor genes known to play a role in hereditary breast cancer, e.g. *BRCA1, TP53* and *PTEN* (light green boxes), as cells lacking the respective functional proteins harbor Warburg-like metabolic features consistent with the CAF phenotype. Well-established environmental cancer predisposition factors (light grey boxes) such as nicotine and ethanol exposure but also chemotherapeutic agents specifically induce the CAF phenotype. Further ROS-generating determinants, e.g. chronic microbial or viral infections, radiation, toxic chemicals, obesity, autoimmune disease, chronic allergen exposure, as well as the aging process itself may set the stage for the evolution of a CAF phenotype, predisposing the organism to tumor development. 2HG, 2-hydroxyglutarate; AMPK, AMP-activated protein kinase; CAF, cancer-associated fibroblast; CAV1, caveolin 1; E2, estradiol; ERα, estrogen receptor alpha; g*BRCA*1m, germline *BRCA1* mutation; GLUT, glucose transporter; g*PTEN*m, germline *PTEN* mutation; GSH, glutathione (reduced form); g*TP53*m, germline *TP53* mutation; HIF1α, hypoxia-inducible factor 1-alpha; IGF1, insulin-like growth factor 1; IGF1R, insulin-like growth factor 1 (IGF-1) receptor; MCT1, monocarboxylate transporter 1; MCT4, monocarboxylate transporter 4; NRF2, nuclear factor E2-related factor 2; OXPHOS, oxidative phosphorylation; PFK2, phosphofructokinase 2; ROS, reactive oxygen species; SIRT, sirtuins; TSC2, tuberous sclerosis complex 2; ULK1, unc-51-like kinase 1; Light yellow boxes, medical/lifestyle interventions; Purple star, oncometabolites.

The more steps towards this process are made, the easier it may be for tumor-initiating cells (i.e. cancer stem cells (CSCs)) to succeed in creating a catabolic stroma environment for their own purpose (Figure 3). This means that any type of non-physiologically elevated prolonged ROS generation, overactive PI3K-AKT-mTOR signaling, and/or favoring of glycolytic flux over OXPHOS may set the conditions for a metabolic switch towards tumorigenesis. As will be explained later in detail, this may include germline *BRCA1* deficiency, *TP53* mutations, and *PTEN* loss. Moreover, activation of proto-oncogenes (e.g. *RAS*, *NFKB1*, *TGFB1*) as well as loss of tumor-suppressor genes (e.g. *BRCA1*) in cancer cells have been shown to be sufficient to induce metabolic reprogramming of the fibroblast compartment *via* ROS generation, and this transformation could be rescued by antioxidants such as N-acetylcysteine [10–12]. Furthermore, well-established environmental modulators of cancer predisposition such as cigarette smoke [13] and ethanol exposure [14] have been demonstrated to specifically induce the CAF phenotype. Since the common basis of all of these contributing factors appears to be endured oxidative stress and chronic inflammation, further environmental or intrinsic determinants causing continuous ROS generation may play a vital role in the emergence of a pro-inflammatory, pro-tumorigenic stromal environment. These include chronic microbial or viral infections, radiation, toxic chemicals, obesity and/or hypercaloric diets, autoimmune disease, and chronic allergen exposure (reviewed in [15]). A majority of the above factors has been associated with disease-related epigenetic changes as well [16], and, interestingly, many of the CAF features are also major contributors to the aging process [17] - age being a clear risk factor for tumor development.

While findings and hypotheses regarding tumor biology have been inconsistent and manifold, it is clearly understood that tumor tissue exhibits overwhelming morphological and physiological intertumoral and intratumoral heterogeneity in its cellular origin, cell surface markers, gene expression profiling, metabolic features, and proliferative as well as metastatic potential [18]. This is likely a reflection of the extremely adaptive nature that allows cancer cells to adjust to individual microenvironmental conditions including alterable nutrient and oxygen supplies. This adaptive quality may also be the cause for pharmacological resistances many tumors appear to acquire during treatment. The cellular phenotype presenting the highest degree of adaptability in a tumor is the CSC-phenotype, which is increasingly made responsible for minimal residual disease, relapse, and metastasis [19–21]. Resistances to redox stress and to chemotherapeutics are considered characteristics of “stemness” [22, 23]. In fact, chemotherapy (CTx) and radiotherapy (RTx) were found to promote a CSC-like phenotype by increasing genotoxic stress and ROS levels [24–26]. CSCs have particular cell surface markers and are capable of sphere formation (e.g. mammosphere growth in breast cancer stem cells), which is one of the hallmark characteristics of stem cells. The expression of genes governing mitochondrial function, autophagy, and lysosome activity has been shown to be upregulated, and a strong reliance on mitochondrial OXPHOS is evident in cells featuring a CSC-like phenotype [27]. Importantly, high mitochondrial mass has recently been introduced as a new feature of the stem-like anabolic phenotype, in which upregulated Wnt/β-catenin signaling promotes the induction of mitochondrial proteins, glycolytic enzymes, the protein synthesis machinery, epithelial-mesenchymal transition (EMT) markers, and increased mitochondrial mass and activity as well as mammosphere formation [28] (Figure 1). Mitochondrial biogenesis is governed by the ERRα-PGC1 signaling pathway, which has been found to enhance mammosphere formation and can be blocked by a specific inhibitor, XCT790, through the suppression of signaling pathways such as sonic hedgehog, TGFβ-SMAD, STAT3, and Wnt-signaling [29]. Induction of a stem-like phenotype in bulk cancer cells has recently been observed by Ning *et al.* in pancreatic cancer cell lines cultured in stem cell medium [30].

Likewise, the induction of a CAF phenotype (Figure 1, Figure 3) in stromal fibroblasts through cancer cells has been described to be mediated by several mechanisms, including miRNAs, TGFβ secretion, and ROS generation promoting HIF1α accumulation [31–33]. The CAF phenotype can, however, also be introduced by CTx [34], comprising proteomic changes in a number of factors including proteins involved in autophagy, inducers of inflammation, metabolic enzymes, antioxidants, and myofibroblast differentiation markers. This generates a catabolic tumor stroma, which strikingly correlates with relapse, metastasis and reduced overall survival. During transformation, fibroblasts undergo myofibroblast differentiation - usually a process initiated upon tissue damage and resulting in fibroblast proliferation and wound healing - and begin to express, among others, α-smooth muscle actin (α-SMA) as a specific myofibroblast marker. The loss of Caveolin 1 (CAV1), which is triggered upon initiation of oxidative stress from adjacent cancer cells and implemented by means of autophagolysosomal degradation, appears to play a major role in the onset of the myofibroblast phenotype [35]. CAV1 loss mediates the upregulation of NFκB and HIF1α activity and ultimately promotes TGFβ signaling, glycolysis, autophagy, and oxidative stress in a feed-forward cycle manner. In CAFs, loss of CAV1 is typically inversely associated with high monocarboxylate transporter 4 (*MCT4*) expression - a marker of lactate efflux and aerobic glycolysis [36, 37], which is upregulated in a HIF1α-dependent manner and shows strict correlation with poor overall survival, particularly so in triple-negative breast cancer (TNBC) patients [38].

The individual cell adaptability and overall inter-tumoral and intra-tumoral heterogeneity makes it extremely difficult for scientists and clinicians to properly treat cancer patients, as each may need highly specific individual handling, and tendencies for relapse and metastases depend on the primary success or failure in eliminating every single tumor cell. Yet, in light of accumulating evidence for what may possibly depict specific overall parallels in tumor metabolism - central pathway alterations and metabolic switches that can consistently be found in the vast majority of tumors - it may be reasonable to consider common, fundamental metabolic root mechanisms of tumorigenesis and, thus, treatment. The aforementioned model of two-compartment tumor metabolism between cancer cells and tumor stroma may be a significant contribution to such attempts and may pose new opportunities for cancer treatment. Current studies are indicative of *both* compartments - that is the catabolic/glycolytic (including CAFs) *versus* the anabolic/oxidative (including CSCs) compartment - contributing to tumor initiation and progression, and also of *both* compartments needing mandatory *parallel* therapeutic targeting in order to prevent relapse and metastatic disease.

Thus far, it is not entirely clear if (i) the reverse Warburg effect may, to a greater or lesser extent, play a role in each tumor, if (ii) strict intertumoral categorization into different metabolic tumor phenotypes, including Warburg-like and reverse-Warburg-like, could be applied, if (iii) these two metabolic phenotypes demonstrate plasticity and adaptability in response to outside triggers and within the process of clonal evolution, or if (iv) even the great mass of bulk cancer cells may comprise completely opposing metabolisms, differing from one cell to the next - some glycolytic-catabolic in the manner of CAFs, others oxidative-anabolic in the manner of CSCs. Therapeutically, however, this may not make a difference as long as each cell could be assigned to either one of the two metabolic compartments and as long as *both* these metabolic extremes were to be targeted simultaneously.

## BREAST TUMOR METABOLOME

To elucidate cancer cell metabolism, a number of tumor metabolome analyses utilizing differing methods have been conducted. However, given the *intra*tumoral heterogeneity in virtually any tumor (including the immediate co-existence of cancer cells, CAFs, normal fibroblasts, macrophages and other immune cells, vascular endothelial cells, adipocytes, neurons and so forth), interpretation of metabolome analyses on a slice of tumor tissue may need to be approached with care. The likelihood of receiving data containing a mixture of *all* of the above cells’ metabolites (some showing “normal” metabolism, some exhibiting Warburg-like and some oxidative metabolism) - which may overall be equational to single metabolite peaks and therefore tamper results - appears to be very high. In order to overcome this burden and to receive unbiased, clearly referable data, tumor tissue may have to be prepared and dissected into separate cell types *via* fluorescence-activated cell sorting (FACS) methods prior to metabolome analyses. Since it is also quite likely that bulk cancer cells immediately adjacent to each other may comprise completely variable metabolic features, single-cell metabolomics analyses could alternatively be applied to CAFs, CSCs, and separate bulk cancer cells. As such analyses are yet waiting to be conducted, we will review current knowledge from tumor metabolome analyses performed thus far.

Despite the metabolic heterogeneity and partly discrepant results, certain metabolic patterns tend to be distinguishable in tumor breast tissue in comparison to normal breast tissue. Whereas the molecular subtypes described from gene expression profiling do not appear to clearly overlap with metabolic profiles, reproducible metabolic signatures do allow the distinction of estrogen receptor-positive (ER^+^) from estrogen receptor-negative (ER^−^) tumors [39–41]. Overall, increased glucose consumption and strong lactate accumulation, even more so in ER^−^ than in ER^+^ breast cancer, is the most typical trait observed in breast cancer and in cancer tissue overall, standing in line with the increased glycolytic activity of both CAFs and cancer cells. At the same time, tumors show an increase in fatty acid synthesis including the immediate consumption of free fatty acids for membranes. An activation of nucleotide and protein biosynthesis for DNA replication and cellular proliferation can be observed. Next to the increase in glycolysis, one can often find additional characteristic catabolic changes, such as drastically increased glutamine consumption in combination with glutamate accumulation, commonly referred to as the “glutamine addiction” phenotype [42]. The glutamate/glutamine ratio (GGR) correlates with estrogen receptor (ER) status in breast cancer (“glutamate enrichment” being found in 56 % of ER^+^ and 88 % of ER^−^ breast cancer, opposing 2.2 % of normal breast tissue), with tumor grading (GGR as a MYC-associated marker of aggressive disease), and with tyrosine metabolism [43].

In terms of more specific markers, a wide range of molecules, enzymes, and metabolite ratios has been suggested as oncometabolites or diagnostic and prognostic biomarkers, many of which can only be touched upon in this review and often still lack functional explanation. For instance, breast tumors generally tend to display high concentrations of taurine, choline, and glycine, the latter of which has been suggested as an erb-b2 receptor tyrosine kinase 2 (*ERBB2*)-associated marker of aggressiveness; also, serine auxotrophy, particularly in aggressive ER^−^ or TNBC, has been observed [40, 44–46]. ER^−^ breast cancer metabolomes generally tend to demonstrate more drastic and characteristic features compared to ER^+^ breast cancer: elevated β-alanine (suggested as the strongest differentiating marker between ER^−^ and ER^+^ breast cancer), an accumulation of 2-hydroxyglutarate (2HG), GSH, branched-chain amino acids, carnitines, and strongly elevated cholines (in particular phosphocholine and phospholipids) combined with elevated glycerol-3-phosphate acyltransferase (GPAM) activity has been detected [39–41, 43, 47]. The uptake of amino acids is usually elevated in breast tumors, particularly that of N-acetyl-aspartate [41]; however, a number of amino acids has been shown to be reduced in several breast cancer cell lines in comparison to a reference breast cell line [48], indicating heightened consumption. Furthermore, an accumulation of kynurenine and a shift within the kynurenine/tryptophan ratio can often be observed, particularly in basal-like breast cancer [40]. Kynurenine is an immunomodulatory metabolic intermediate generated during tryptophan degradation and NAD^+^/nicotinic acid biosynthesis. Lactate, physiologically produced excessively under hypoxic conditions, acts immunomodulatory as well, fuels angiogenesis and metastasis, and constitutes a major contribution to establishing the relationship between tumor cells and tumor microenvironment [6, 49]. Prostaglandins, which are derived from aberrantly increased cyclooxygenase-2 (COX2) in breast tumors and which function as oncogenic lipid messengers [50–52], link breast cancer to inflammatory processes. A high ratio of glycerophosphocholine to phosphocholine (GPC/PCho) has been proposed as a marker of basal-like and luminal B breast tumors but has yielded discrepant results (reviewed in [53]). In turn, the cytidine-5-monophosphate/pentadecanoic acid ratio has been demonstrated to be a fairly reliable discriminator between cancerous and normal tissue, detecting cancer with a sensitivity of 94.8 % and a specificity of 93.9 % [54].

## THE INTERPLAY BETWEEN 2HG, MYC, AND GLUTAMINE CATABOLISM

**Figure 4:**
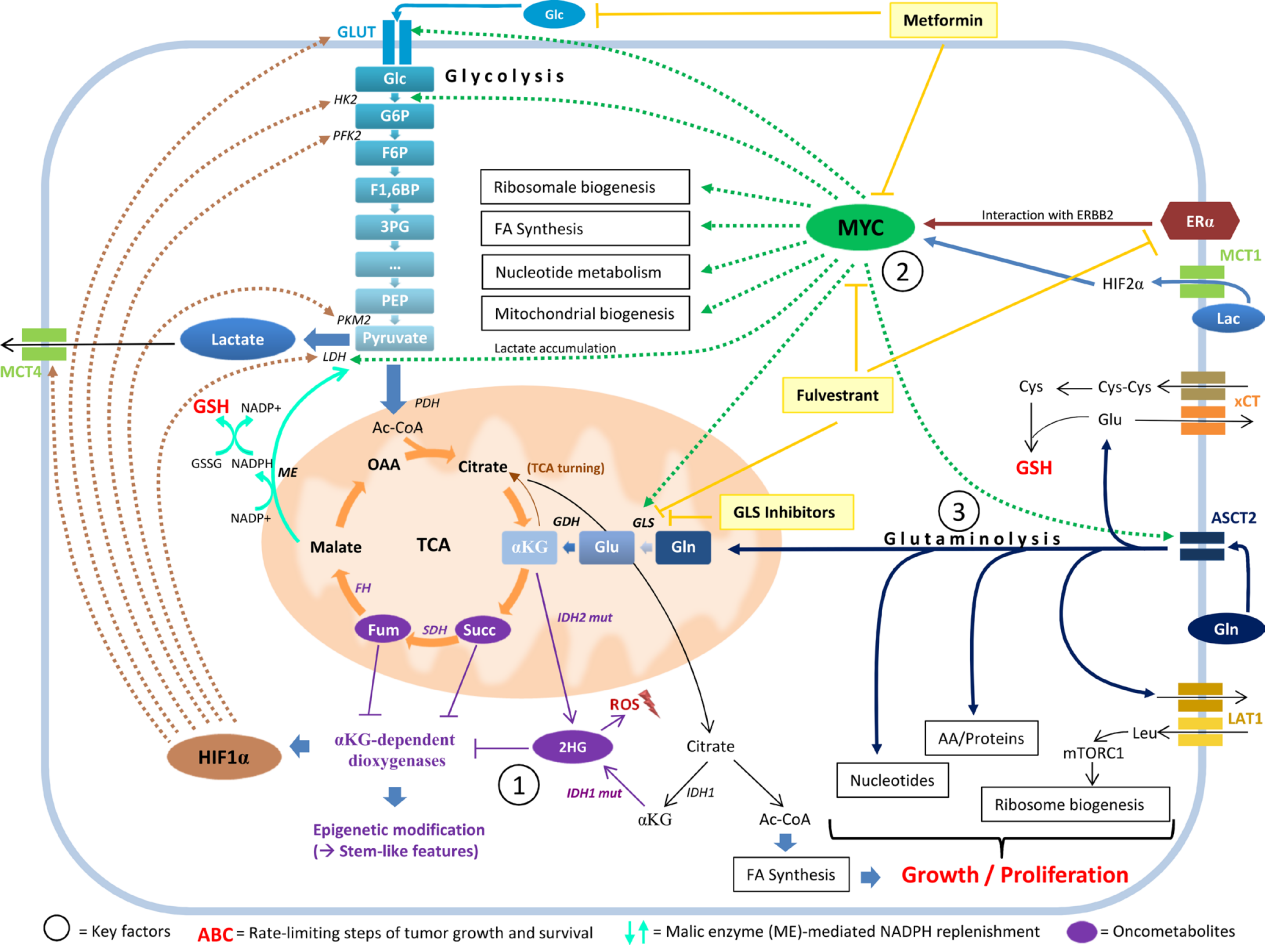
The interplay between 2HG, MYC, and glutamine catabolism MYC pathway activation, glutaminolysis, and the oncometabolite 2HG stand in a functional relationship to each other and are associated with stem-like features in cancer cells. 2HG accumulation (Key factor 1) inhibits αKG-dependent dioxygenases, which results in stabilization of HIF1α and in extensive epigenetic alteration. These methylation-specific changes cause a block of cell differentiation and an increase in the expression of stem and progenitor cell markers. 2HG accumulation is often associated with MYC pathway activation (Key factor 2) in breast cancer cells. MYC maintains stem cell pluripotency and simultaneously influences major metabolic pathways implicated in overactive tumor cell metabolism, including mitochondrial biogenesis. *MYC* expression is affected by the interaction of ERα with ERBB2 and strongly influences glutamine metabolism by regulating glutaminase expression. A metabolic switch towards activated glutaminolysis is mediated by extracellular lactate uptake *via* MCT1, stabilizing HIF2α and transactivating MYC, which subsequently triggers the expression of glutamine transporter ASCT2 and of glutaminase (GLS). Glutaminolysis (Key factor 3) contributes to cancer cell metabolism by fueling OXPHOS through the replenishment of TCA cycle intermediates, supplying building blocks for proliferation, replenishing GSH pools for ROS-scavenging (which involves the xCT cystine/glutamate antiporter for cysteine acquisition and the generation of NADPH by malic enzyme to reduce GSSG back to GSH), coupling glutamine export with leucine-import (which mobilizes mTORC1 and stimulates ribosome biosynthesis), and promoting 2HG accumulation *via* its precursors glutamate and αKG. 2HG, 2-hydroxyglutarate; 3PG, 3-phosphoglycerate; AA, amino acids; Ac-CoA, acetyl coenzyme A; ASCT2, ASC amino-acid transporter 2; αKG, alpha-ketoglutarate; Cys, cysteine; Cys-Cys, cystine; ERα, estrogen receptor alpha; ERBB2, erb-b2 receptor tyrosine kinase 2; F1,6BP, fructose 1,6-bisphosphate; F6P, fructose 6-phosphate; FA synthesis, fatty acid synthesis; FH, fumarate hydratase; Fum, fumarate; G6P, glucose 6-phosphate; GDH, glutamate dehydrogenase; Glc, glucose; Gln, glutamine; GLS, glutaminase; Glu, glutamate; GLUT, glucose transporter; GSH, glutathione (reduced form); GSSG, glutathione disulfide (oxidized form); HIF1α, hypoxia-inducible factor 1-alpha; HIF2α, hypoxia-inducible factor 2-alpha; HK2, hexokinase 2; IDH1/2, isocitrate-dehydrogenase 1/2; IDH1/2 mut, isocitrate-dehydrogenase 1/2 mutant; Lac, lactose; LAT1, L-type amino acid transporter 1; LDH, lactate dehydrogenase; Leu, leucine; ME, malic enzyme; MCT1, monocarboxylate transporter 1; MCT4, monocarboxylate transporter 4; mTORC1, mechanistic target of rapamycin complex 1; NADP^+^/NADPH, nicotinamide adenine dinucleotide phosphate (oxidized and reduced form); OAA, oxaloacetate; PDH, pyruvate dehydrogenase; PEP, phosphoenolpyruvate; PFK2, phosphofructokinase 2; PKM2, pyruvate kinase isozyme M2; ROS, reactive oxygen species; SDH, succinate dehydrogenase; Succ, succinate; TCA, tricarboxylic acid cycle; xCT/SLC7A11, solute carrier family 7 (xc- system), member 11, cystine/glutamate transporter; Light yellow boxes, medical interventions.

Metabolic diversity between individuals originates from exogenic as well as endogenic influencing factors, including germline or somatic mutations, and the resulting metabolic state may or may not have a decisive impact on disease predisposition in humans. More precisely, the question as to whether metabolic alterations merely represent byproducts from oncogenesis, whether they function as “reactive” mediators antagonizing the oncogenic process, or whether they could indeed be etiologically seminal for tumorigenesis - quasi acting as oncometabolites - will need to be further addressed in detail. However, in the context of rare hereditary metabolic diseases, termed “inborn errors of metabolism” (IEMs), metabolic changes *have* been shown to be capable of initiating malignant transformation [55]. In this regard, the focus has primarily been set on three oncometabolites - namely fumarate, succinate, and MYC-associated 2HG - the latter of which will be the focus of the below paragraph (Figure 4, Key factor 1). These oncometabolites accumulate in the presence of defective mitochondrial tricarboxyl acid (TCA) cycle enzymes fumarate hydratase (FH), succinate-dehydrogenase (SDH), and isocitrate-dehydrogenase 2 (IDH2) as well as the cytosolic isocitrate-dehydrogenase 1 (IDH1). Their accumulation results in competitive inhibition of alpha-ketoglutarate (αKG)-dependent dioxygenases and, among other consequences, leads to extensive epigenetic alteration as well as HIF1α stabilization, which raises the expression levels of glycolytic enzymes. Additionally, fumarate modifies the function of thiol-containing compounds such as glutathione.

Notably, 2HG has emerged as a main focus of interest, particularly in the context of glioma and leukemia, which tend to accumulate 2HG by means of *IDH1* or *IDH2* mutations [56, 57]. It is important to note the enantiomer-specific effect of D-2-hydroxyglutarate (D-2HG) *versus* L-2-hydroxyglutarate (L-2HG) - both having been implicated in tumorigenesis - whereas ‘2HG’ usually refers to the sum of the two. As not all data distinguishes between D-2HG and L-2HG, we will refer to the cited data in the original manner of the study. Breast tumors, in comparison to normal breast tissue, have been shown to comprise high levels of 2HG - more than 200-fold in ER^−^ breast cancer (basal-like/mesenchymal tumors showing the highest concentrations) and to a smaller (around 20-fold) extent in ER^+^ breast cancer [40]. In the context of breast tumors, however, mutations in IDH enzymes have not been identified, which is why alternative mechanisms of 2HG accumulation are being discussed (e.g. alcohol dehydrogenase, iron containing 1 (*ADHFE1*) overexpression or D-2-hydroxyglutarate dehydrogenase (*D2HGDH*) downregulation) [41]. Intracerebral 2HG injection in rats (both D-2HG and L-2HG) has resulted in increased oxidative damage on lipids, proteins, and DNA, paralleled by the suppression of antioxidative defense mechanisms [58, 59]. As mentioned above, both D-2HG and L-2HG have been shown to inhibit αKG-dependent dioxygenases, including prolyl hydroxylase domain (PHD) enzymes (which usually help degrade HIF1α), histone demethylases, and the ten-eleven translocation (TET) enzyme family [60]. The latter hydroxylases 5-methyl-cytosine (5mC) to 5-hydroxy-methyl-cytosine (5hmC), causing a dramatic shift in the 5hmC/5mC ratio and a genome-wide DNA hypermethylation phenotype [57, 60, 61]. Notably, these changes in histone and DNA methylation have been shown to cause a block of cell differentiation and an increase in the expression of stem and progenitor cell markers, indicating a switch to a stem-like phenotype in cancer cells (Figure 4, Key factor 1). Consistent with this, a striking overlap of 2HG accumulation and MYC pathway activation (Figure 4, Key factor 2) can be observed [41]. As MYC is known to be essential in maintaining stem cell pluripotency [62] and as it antagonizes re-differentiation of neoplastic cells into typical tissue-specific cells [63], these findings appear to be of utmost relevance for tumorigenesis. In comparison to other oncogenes, MYC is unique in the way that it simultaneously influences all cellular pathways that cumulatively contribute to overactive tumor cell metabolism: ribosome biogenesis, glycolysis, glutaminolysis, fatty acid synthesis, nucleotide metabolism, lactate accumulation, and - possibly most suggestive of a stem-like phenotype - mitochondrial biogenesis [64, 65]. This wide range of functions is reflected in a strong metabolic signature in tumors that harbor MYC pathway activation [41]. The MYC pathway even provides means for bypassing PI3K signaling, which is why resistance to PI3K pathway inhibitors often goes alongside *MYC* amplification [66, 67]. About 40-45 % of all breast tumors exhibit *MYC* overexpression [68], which is associated with unfavorable prognostic markers. MYC is a target of WNT/β-catenin signaling, which induces epigenetic repression of *BRCA1* expression by means of snail family zinc finger 2 (SNAI2) and is associated with basal-like breast cancer and EMT, including metastasis [69]. Possibly offering the most important contribution to a major metabolic switch in tumor tissue, MYC strongly influences glutamine metabolism (Figure 4, Key factor 3) by regulating glutaminase (*GLS*) expression through miRNAs [70, 71]. A subgroup of the most 2HG-rich breast tumors showed a specific methylation pattern characterized by hypomethylation of the *IDH2* locus and *IDH2* overexpression; this subtype was more prevalent in patients of Afro-American descent with ER^−^, stem cell-like breast cancer, *GLS* overexpression, and reduced overall survival [41]. As *MYC* overexpression increases, and *MYC* knockdown decreases intracellular 2HG levels, but 2HG levels also decrease following GLS inhibition [41], a functional relationship between MYC, glutamine catabolism and 2HG accumulation is likely. Recent findings have highlighted a possible mechanism which may enable cancer cells to induce a metabolic switch towards activated glutaminolysis [72] (Figure 4, upper right corner): extracellular lactate uptake by means of monocarboxylate transporter 1 (MCT1) first stabilizes HIF2α, which then transactivates MYC in a pseudohypoxic response. This subsequently triggers the expression of glutamine transporter *ASCT2* and of *GLS*, promoting glutamine uptake and catabolism. Glutamine catabolism, in turn, contributes to cancer cell metabolism in multiple ways, possibly provoking “glutamine addiction” in cancer cells (Figure 4, Key factor 3): (i) Glutamine fuels OXPHOS [73] and, thus, the very metabolic feature cancer cells - in contrast to CAFs - appear to depend on. Inhibition or knockdown of GLS has resulted in reduced OXPHOS, leading to an arrest in cell proliferation and activated mitochondrial apoptosis [73]. The mechanism by which glutaminolysis exerts its effect on OXPHOS is thought to mainly involve replenishment of TCA cycle intermediates by converting glutamine to glutamate and further to αKG; this way glutamine serves as the major carbon source for the TCA cycle. (ii) Glutamine supplies building blocks for cell proliferation by serving as a nitrogen source for nucleotides and amino acids and by supporting fatty acid synthesis through a process termed TCA turning (a partly reverse running TCA cycle) [74, 75]. (iii) Glutamine promotes the replenishment of reduced glutathione pools for ROS scavenging and subsequent escape from ROS-induced cell death, helping tumor cells to survive. This mechanism involves (a) the xCT (SLC7A11) antiporter for cysteine/glutamate exchange (Figure 4, right rim), which is expressed on one third of TNBC *in vivo* [76] and accounts for cysteine acquisition and subsequent use in GSH generation (reviewed in [77]). GSH consists of only three amino acids, these being glycine, cysteine, and glutamate, the last of which is directly synthesized from glutamine. Moreover (b), glutaminolysis is a means for NADPH generation *via* the conversion of glutamine to pyruvate by malic enzyme, which is necessary to reduce the oxidized form of glutathione - glutathione disulfide (GSSG) - back to its reduced form - GSH -, and thus replenish GSH pools [78] (Figure 4, turquoise arrows). (iv) Intracellular glutamine levels are crucial for quick proliferation in the way that glutamine export is coupled with an import of leucine, which mobilizes mechanistic target of rapamycin complex 1 (mTORC1) and stimulates ribosome biosynthesis [64]. (v) Glutamine catabolism is also involved in the aberrant accumulation of 2HG since αKG, glutamate, and glutamine serve as precursors for this oncometabolite. The aforementioned link between 2HG and a de-differentiation into a stem-like phenotype thus appears to complement a feed-forward cycle promoting CSC maintenance.

Glutaminase inhibitors have already been used successfully in preclinical studies inhibiting the proliferation of TNBC but not of ER^+^ breast cancer cell lines [79, 80]. By means of “glutamine deprivation”, *MYC* expression can be reduced in cell cultures and apoptosis can selectively be triggered in *MYC*-transformed cells [81]. As *MYC* expression can also be significantly influenced by the interaction of estrogen receptor alpha (ERα) with ERBB2, and as MYC-associated glutamine dependence has been linked to aromatase inhibitor (AI) resistance in ER^+^ breast cancer, fulvestrant - an ERα downregulator that also inhibits *MYC* expression and glutaminase - has been suggested as a therapeutic agent in AI-resistant ER^+^ breast tumors [82] and may experience future repurposing for further indications (Figure 4, upper right corner).

## METABOLIC SIGNATURES INTRODUCED BY MUTATIONAL LANDSCAPE

Studies focusing on metabolic and pathway analyses in breast cancer cell lines either harboring (knock-in) or lacking (knock-out) a specific functional wild-type tumor suppressor gene can help elucidate metabolic changes associated with mutations in single genes or pathways. Differential studies on the metabolome of non-tumorous breast tissue derived from patients carrying a specific mutation in a breast cancer predisposition gene *versus* non-mutation carriers can also help elucidate metabolic alterations in those cells. Genes such as *BRCA1*, *BRCA2*, and *TP53*, but also *PTEN, PIK3CA, CHEK2, RAD51C,* and *ATM* are of major interest in this regard. Strikingly, results indicate a close relationship between tumor suppressor genes typically mutated in breast cancer and Warburg-like metabolism in those cells lacking the functional protein. In a germline setting, it is conceivable that a reduced dosage of functional protein or a dominant-negative effect of the mutated over the wild-type protein may not only drive tumor-initiating cells in acquiring a malignant phenotype, but may also result in a Warburg-like metabolic switch in the stromal environment. This, in turn, may predispose the organism to tumor development by setting the stage for the evolution of a CAF phenotype (Figure 3).

For instance, mutations in genetic components of the PI3K-AKT-mTOR-PTEN pathway usually result in strong basal activation of mTOR signaling, which is one of the most characteristic features of the CAF phenotype. Up to 44 % of all breast cancer subtypes bear PI3K pathway aberrations such as *PIK3CA, PIK3R1, AKT1,* and *PTEN* mutations, in particular luminal A (53.4 %) and *ERBB2*-enriched (47 %) subtypes, although much less common in the basal-like (10 %) subtype [83]. Research continuously focuses on this signaling pathway, as its activation appears to be an overall prerequisite for tumor formation. The pathway acts in an essential pro-proliferative and anti-apoptotic manner, stimulating protein and fatty acid synthesis, activating glycolysis, and promoting lactate efflux [84, 85]. Stimulation of glycolytic flux is accomplished by the upregulation of hexokinase 2 (*HK2*) and *HIF1A* expression mediated by AKT1 and mTORC1, respectively. Loss of *PTEN*, which physiologically counteracts PI3K function by dephosphorylating phosphatidylinositol (PI) 3,4,5-triphosphate to PI-4,5-bisphosphate, additionally impairs mitochondrial function *via* the interplay between PTEN and TP53 [86].

TP53 mutations, in turn, which are amongst the most frequent mutational events in cancers and can be found in about 37 % of all breast tumors and in up to 80 % of basal-like and *ERBB2*-enriched tumors [83], have also been demonstrated to cause the metabolic switch towards glycolysis in a Warburg-like manner (Figure 3). This mechanism involves the downstream TP53 target genes TP53-induced glycolysis and apoptosis regulator (*TIGAR)* and synthesis of cytochrome c oxidase-2 (*SCO2)*. Whereas SCO2 regulates the cytochrome c oxidase complex, which is critical in OXPHOS [87], TIGAR operates as a fructose-2,6-bisphosphatase, resulting in the inhibition of glycolysis and the promotion of the pentose phosphate pathway (PPP) [88, 89]. Elevated NADPH levels due to activated PPP promote GSH synthesis and, thus, help to scavenge intracellular ROS, inhibiting autophagy [90, 91]. Loss of TIGAR, in turn, raises intracellular ROS levels and induces autophagy, in line with the autophagic CAF phenotype. In addition, wild-type TP53 is known to inhibit mTORC1 and its downstream targets, ultimately impairing protein synthesis and proliferation. In Li-Fraumeni syndrome (MIM 151623), which is a disorder caused by heterozygous *TP53* germline mutations predisposing the subject to multiple malignancies including breast cancer, it is conceivable that the aforementioned major impact on energy metabolism, pro-proliferative signaling, and regulation of cellular survival mechanisms may result in stromal tissue that assists tumor initiating cells by presenting an environment according to their needs. Notably, one particular finding may further substantiate an impact of *TP53* loss on metabolic coupling in tumors. TP53 is known to repress monocarboxylate transporter 1 (*MCT1*) expression, while *TP53* deficiency results in elevated *MCT1* expression [92]; however, the consequences of altered *MCT1* expression levels appear to depend on the metabolic setting. Under hypoxic conditions, as mimicked in CAFs, elevated *MCT1* expression due to loss of *TP53* promotes lactate *export*, whereas under conditions of additional glucose deprivation - as may be simulated in cancer cells due to heightened energy demands - *MCT1* expression promotes lactate *import*. Through this differentiated mechanism, cancer cells could profit greatly from the lactate efflux originating from adjacent CAFs.

Similar phenomena in terms of creating a “tumor-friendly” environment in stroma tissue have recently been observed concerning the tumor suppressor gene *BRCA1* (Figure 3). *BRCA1*-associated breast cancer is often triple-negative or basal-like, which commonly confers poor prognosis [93]. Although mainly known for its role in homologous recombination DNA repair, BRCA1 has increasingly been attracting attention for various further functions, including its extensive role in cellular energy metabolism and in the regulation of oxidative stress [94, 95]. A *BRCA1*-mutated breast cancer cell line transfected with wild-type *BRCA1* has displayed the reversal of Warburg-like metabolic features, including an activation of OXPHOS and an impairment of glycolytic flux *via* the inhibition of the expression of genes that all play major roles in glycolysis (e.g. *SLC2A1, HK1, HK2, PFKFB3,* and *LDHA*) [94]. BRCA1 also interacts with HIF1α, AKT1, MYC, and TP53, which have well-established roles in the regulation of glycolysis. Changes in lipid metabolism point to increased beta-oxidation and decreased fatty acid synthesis by means of acetyl-CoA-carboxylase-alpha (ACCA) inhibition through BRCA1 *via* stabilization of the phosphorylated and inactive form of ACCA, pACCA; free fatty acids and ketone bodies are consumed, and the generated acetyl-CoA may subsequently be fed into the TCA cycle [40, 94, 96]. CoA, acetyl-CoA, and several acylcarnitines are positively associated with *BRCA1* mRNA levels, while various lipids (long chained fatty acids and membrane components) and amino acids are inversely correlated with *BRCA1* transcription levels [40]. All of the above associations are consistent with a strong antagonizing impact of BRCA1 on Warburg-like metabolism.

Another interesting relationship exists between BRCA1 and nicotinamide-adenine-dinucleotide (NAD^+^): *BRCA1* knockdown results in elevated NAD^+^ levels, whereas *BRCA1* mRNA levels conversely increase with available NAD^+^ [97]. NAD^+^ is a weak competitive inhibitor of complex I of OXPHOS (NADH dehydrogenase) [98]. In the stroma of *BRCA1* mutation carriers, low levels of BRCA1 resulting in elevated NAD^+^ supplies may thus exert an inhibitory effect on OXPHOS, contributing to the CAF phenotype. As Poly(ADP-ribose)-Polymerase (PARP) enzymes also use NAD^+^ as a substrate for their functioning in base excision repair, and as PARP inhibitors - suggested specifically for the treatment of *BRCA1/2*-mutated cancers - raise the pool of available NAD^+^, this association may deserve particular attention.

Moreover, BRCA1 greatly influences endocrine parameters. Aromatase expression is inhibited by BRCA1 in a manner similar to TP53 (Li-Fraumeni patients exhibit raised aromatase expression [99]), therefore limiting estradiol (E2) production [100]. BRCA1 impairs the transcriptional activity of ERα, and overexpression of *BRCA1* subsequently inhibits the expression of 90 % of estrogen-inducible genes [101]. Furthermore, BRCA1 reduces expression levels of insulin-like growth factor 1 receptor *(IGF1R)* and its ligand *IGF1* [102, 103]. Accordingly, *BRCA1* germline mutation carriers are exposed to higher serum E2 levels (particularly in luteal phase) [104, 105], exhibit a higher activity of ERα and elevated *IGF1R* expression levels in estrogen-stimulated cells [106], and show intratumoral accumulation of IGF1 [107] combined with a decrease in IGF1 serum concentrations (likely due to increased binding to IGF1R) [100]. While an excess supply of E2 and also IGF1/2 upregulates *BRCA1* expression in a compensative manner in *BRCA1* wild-type cells [108, 109], this mechanism is impaired in mutation carriers. Consequently, the PI3K-AKT-mTOR pathway is stressed in multiple ways in *BRCA1*-deficient cells: E2 directly activates PI3K and AKT1, IGF1 activates the pathway by binding to IGF1R, and ERα provokes the phosphorylation of AKT1, while the inhibition of the latter by BRCA1 is hampered [110–112]. Phosphorylated and activated AKT1, in turn, stimulates the expression of ERα in a feed-forward mechanism [113]. If, in addition to this, insulin levels happened to be raised (e.g. in a pre-diabetic metabolic state), this would constitute further direct activation of the pathway as well as an increase in E2 levels [114].

Furthermore, antioxidative signaling pathways, including glutathione metabolism, are extensively upregulated by BRCA1. Direct interaction of BRCA1 with NRF2 - the “master regulator of antioxidant responses”, which is activated and stabilized by this interaction - induces the expression of various antioxidant enzymes that protect the cell from ROS and therefore prevent oxidative DNA damage [115]. NRF2 represents a Janus face in tumor biology [116]: while, on the one hand, helping to prevent malignant transformation in the first place (*Nrf2* deficiency results in tumor initiation in mice [117], and loss of NRF2 activity due to *BRCA1* deficiency has been suggested to contribute to (early-onset) carcinogenesis [95]), excessive NRF2 activation helps tumor cells, on the other hand, to escape apoptosis by upregulating ROS-scavenging antioxidant signaling such as glutathione replenishment. Indeed, *NRF2* is known to be strongly upregulated in many tumors and to be associated with aggressive disease and bad prognosis [7]. Gorrini *et al.* [112] addressed this issue and created an interesting hypothesis concerning the E2-associated tissue specificity of *BRCA1*-deficient tumors, as follows. Estrogen abundance induces NRF2 accumulation in a PI3K-AKT-mTOR pathway-dependent manner (PI3K-pathway inhibition prevents NRF2 activation). In a physiological setting, this represents a reasonable mechanism, since the generation of estrogen metabolites (e.g. 4-OHE1/E2 or reactive estrogen quinones resulting from oxidation) can induce oxidative DNA damage by forming mutagenic depurinizing adenine/guanine-estrogen-DNA-adducts [118]. Under normal circumstances, an NRF2 accumulation would subsequently be followed by an increase in *BRCA1* expression [119], initiating the repair of DNA damage parallel to ROS scavenging and cell survival. This is obviously not possible in *BRCA1*-deficient cells (and may additionally be impaired in wild-type *BRCA1* cells by mechanisms such as epigenetic repression or NAD^+^ depletion). Hence, PI3K hyperactivation (either by means of constant stimulation, or by means of *PTEN* deficiency, which can often be found in *BRCA1*-mutated tumors [120]) *coupled with* E2 stimulation (which is tissue-specific and generally elevated in *BRCA1* mutation carriers) may lead to NRF2 accumulation in *BRCA1*^−/−^ pre-tumor cells (germline first hit and somatic second hit), helping the cell to escape ROS-induced cell death. Combined with the lack of BRCA1 DNA repair efficiency and resultant genomic instability, this may ultimately induce malignant transformation. Tissue types that are not stimulated by estrogens for the lack of appropriate receptor abundance would not be affected by this mechanism and, thus, were to induce ROS-associated cell death in the case of somatic second hit and randomly generated *BRCA1*^−/−^ cells.

Finally, BRCA1 has been identified as an important player in autophagy, in the sense that *BRCA1* deficiency promotes Beclin 1-dependent autophagic pathways in response to metabolic stresses (e.g. serum starvation or endoplasmatic reticulum stress) [121]. In *BRCA1*-deficient cancer cells, this stress-mitigating mechanism helps to prevent cell death, supporting cancer cell survival. In stromal cells of *BRCA1* mutation carriers, in turn, these findings point to an innate enhancement of autophagic pathways, which is in agreement with the CAF phenotype.

In conclusion, BRCA1 impairment can be expected to result in Warburg-like metabolism, activation of PI3K signaling, elevated ROS signaling, impaired antioxidant response, and enhanced autophagy - the very features of the CAF phenotype. As silencing of *BRCA1* expression by means of promoter hypermethylation has also been suggested as an important event in the pathogenesis of sporadic breast cancer disease [122–124], particularly so in TNBC, these findings may have broad implications beyond hereditary breast cancer pathogenesis.

## BLOOD-BASED METABOLOME ANALYSES AND BIOMARKERS

Besides focusing on tumor tissue and adjacent non-tumorous tissue, it also appears promising to metabolically analyze peripheral white blood cells as well as correlated serum or plasma samples of (i) hereditary breast cancer patients, classified by the affected gene, *versus* (ii) corresponding mutation carriers who have not (yet) developed cancer, and (iii) a healthy control collective without history of familial breast cancer. Similar to the influence single nucleotide polymorphisms (SNPs) are known to exert on blood metabolite levels [125, 126], systemic metabolic changes resulting from specific germline mutations, possibly setting the stage for tumor formation, are conceivable and may be reflected in these particular analyses. A comparison between diseased *versus* non-diseased individuals affected by the same germline mutation would be of particular value, preferably focusing on individuals from the same family lineage in order to reduce metabolic noise stemming from overall genetic variability. Likewise, systemic metabolic changes that may predispose an individual to breast cancer development in a sporadic setting and which may be reflected by changes in biofluids or peripheral blood cells are of high interest and have partly been addressed already. At this point, however, it is difficult to securely attribute the metabolic variation detected in serum or plasma of cancer patients either to changes having *predisposed* the individual to tumor formation in the first place, or to changes being *the result* of tumor formation. Longitudinal epidemiologic studies will help elucidate this matter. For environmental factors known to contribute to sporadic breast cancer risk, e.g. smoking, alcohol consumption, or BMI, a strong correlation between an exposure to these factors and blood metabolites has already been confirmed [127–131].

As for finding adequate biomarkers to predict disease occurrence and disease status, no single marker but rather specific sets of markers, and multiple versions thereof, have been suggested from serum and plasma metabolome analyses on breast cancer patients independent of mutational status. While these marker sets vary substantially between each other, they do consistently include modifications within glycolysis parameters, phospholipid metabolism, fatty acid metabolism, and amino acid metabolism (reviewed in [53]). One study by Asiago *et al.* [132] presented a set of 11 markers (namely formate, histidine, prolin, choline, tyrosine, 3-HB, lactate, glutamic acid, N-acetyl-glycine, 3-hydroxy-2-methyl-butanoic acid, and nonanedioic acid) being able to predict recurrent breast cancer disease with a sensitivity of 86 % and a specificity of 84 %. In about half of these cases, this prediction could be made more than a year prior to clinical diagnosis for relapse. Similar advances were achieved in a study by Tenori *et al.* [133], in which lower levels of histidine and higher levels of glucose, lactate, tyrosine, and lipids were able to predict metastatic disease in 83.7 % of cases, which were predominantly ER^−^. Likewise, Jobard *et al.* [134] identified a set of nine metabolites (histidine, acetoacetate, glycerol, pyruvate, N-acetyl glycoproteins (NAC 1 and 2), mannose, glutamate, and phenylalanine) associated with advanced metastatic disease. Three fatty acids (C22:0, C24:0, and C18:2n6 - namely behenic acid, lignoceric acid, and linoleic acid, respectively) were identified to be significantly reduced in plasma of premenopausal and postmenopausal breast cancer patients [135]. Yet another study suggested mainly two markers, lysophosphatidylethanolamine and ceramide, to play an important role in invasive ductal carcinoma [136]. Notably, low circulating levels of the amino acid aspartate have recently been correlated with breast cancer occurrence, and this association was specific to breast tumors as opposed to other malignancies such as gastric or colorectal cancers [137]. Since breast tumors tend to accumulate high intracellular levels of aspartate, increased tumor uptake has been suggested to attribute for low circulating levels of this amino acid. In conjunction with this, it is important to note that, in addition to heightened uptake, aspartate biosynthesis has been identified to be intrinsically elevated in cancer cells and is discussed as one of the chief requirements underlying excessive proliferation as well as redox balance for survival [138, 139].

To the best of our knowledge, untargeted metabolome analyses have not yet been specifically conducted on serum/plasma samples of *BRCA1* or *BRCA2* mutation carriers. Serum proteomic analyses on *BRCA1* mutation carriers have, however, revealed great differences between the protein expression profile of (i) individuals who developed a tumor within the next 3 years, possibly harboring a tumor already at baseline, (ii) those who stayed tumor-free within 7 years of follow-up (until trial closure), and (iii) a cohort of sporadic breast cancer patients; in contrast, accurate discrimination was not possible between healthy controls *versus* tumor-free *BRCA1* mutation carriers [140]. Another more recent proteomic study [141] on *BRCA1* mutation carriers of a specific founder mutation suggested a *BRCA1*-specific proteomic profiling signature and the downregulation of the protein gelsolin as a promising biomarker differentiating between diseased and non-diseased individuals.

Without question, it would be desirable to identify diagnostic and prognostic biomarkers of even superior sensitivity and specificity to the above metabolite ratios in serum/plasma of breast cancer patients (mutation carriers as well as non-mutation carriers). Importantly, these need to be suitable for clinical use and will have to prove reliable in the long run, reducing false-positive and false-negative results to a minimum, which is of particular relevance for such a widespread disease of high overall prevalence.

## PREVENTIVE AND THERAPEUTIC MEASURES

For preventive action regarding mutation carriers in particular but also the general public, deep knowledge of aberrant metabolic signaling and enzyme/substrate modification will give important clues about etiologically significant factors in need for targeting on a long-term basis. Ideally, altered signaling resulting from reduced expression of tumor suppressor genes could be antagonized on a metabolic level downstream of genomic and transcriptomic events, balancing the aberration's consequences. By identifying appropriate, targeted means of intervening in misbalanced biochemical signaling patterns (e.g. *via* drugs such as small molecular PI3K-AKT-mTOR inhibitors, glutaminase inhibitors, fulvestrant, metformin, but possibly also by means of glutamine and glucose restriction, specific substrate substitution, antioxidant modification, and general lifestyle changes), one may eventually get down to decreasing tumor rates in genetically predisposed individuals. This includes *BRCA1/2* mutation carriers, whose sole option for actively reducing tumor burden currently involves risk-reducing surgery (mastectomy, adnexectomy).

At the same time it is indispensable to find suitable therapeutic means once tumors have arisen. In particular, this concerns TNBC patients for whom, as of date, no standard established treatment line exists due to the absence of specific target receptors. TNBC patients account for about 15 % of all breast cancer cases [93], and about 10-15 % of these harbor germline *BRCA1* mutations [142]. According to genomic profiling, TNBC can further be classified into two major subgroups, *BRCA1*-like and non-*BRCA1*-like. A study by Severson *et al.* [143] recently revealed that about 55 % of the analyzed TNBC samples could be classified as *BRCA1*-like, including around 15 % exhibiting *BRCA1* promoter hypermethylation and 10 % harboring a *BRCA1* germline mutation. These *BRCA1*-like tumors were found to frequently harbor somatic *TP53* mutations, whereas non-*BRCA1*-like tumors frequently harbored mutations in PI3K pathway components such as *PIK3CA*. This implies that separate subgroups within TNBC may need entirely different treatment (e.g. PARP inhibitors for *BRCA1*-like tumors and PI3K pathway inhibitors for non-*BRCA1*-like tumors). Moreover, a few other studies concerning TNBC have recently identified a number of mutated genes, particularly DNA repair genes and cell cycle regulators, in *BRCA1/2* mutation-negative TNBC [144–146]. Thus, further splitting of the two major subgroups into additional sub-categories appears likely and may reveal more distinct metabolic information and therapeutic options.

Within the context of personalized medicine, approaching each tumor, each patient, and each mutation carrier on an individual basis according to metabolomic and genomic profiling will eventually enable customized preventive and therapeutic measures. Whereas gene-therapeutic means - still rather utopic for the time being - would certainly depict the most causal treatment for mutation carriers, metabolic modification occurring downstream of the genomic level should attempt to tackle biochemical imbalances as far up the root as possible. The further downstream the intervention takes place, the more likely it will be that only part of the matter is solved and that the tumor cell finds a way to bypass the intervention by means of metabolic adaptation, thus, acquiring resistance. However, in order to fully evaluate, assess, and lastly benefit from each tumor's individuality, it is indispensable to thoroughly comprehend root metabolic mechanisms of tumor cell proliferation, cell growth, and cell survival, as well as rate-limiting steps thereof (Figure 4, bright red text). This will allow creating a more complete overall picture of tumor biology, in which individual aberrations can subsequently be assessed in a differentiated manner.

These root metabolic mechanisms and their rate-limiting steps require in-depth discussion and will likely be subject of ongoing future debate and research. In terms of (i) tumor cell *survival*, it appears reasonable to suggest the ROS-scavenging potential of tumor cells being one of the most essential rate-limiting steps. Some tumors, particularly ER^−^ ones, may achieve this goal by upregulating MYC - either through *MYC* amplification or by means of *MYC* overexpression (e.g. due to great amounts of lactate import from adjacent stroma cells) -, which should result in activated glutaminolysis and subsequent GSH production, conferring a “glutamine addiction” phenotype. Other tumors, mainly ER^+^ ones, may achieve the same goal by the simple upregulation of estrogen receptors in an estrogen-rich setting, leading to (a) NRF2 accumulation in the presence of an activated PI3K pathway and, thus, elevated antioxidant response, and to (b) *MYC* expression [147] through a process involving an upstream enhancer and the activator protein 1 (AP-1) transcription factor, leading to similar consequences as described above. The effect is the same. However, these mechanisms may be adaptable and even overlap to some extent, which would make therapeutic targeting even more challenging. For instance, once ER^+^ tumor cells find themselves in an estrogen-deprived environment due to antiestrogen therapy such as tamoxifen, some of these cells - especially CSCs - might theoretically be capable of adapting to this challenge by switching to an alternative way of accumulating GSH (e.g. glutamine consumption). On the other hand, once glutamine-dependent tumors were to be targeted by glutaminase inhibition, they might adapt to alternative ROS scavenging mechanisms, such as NRF2 upregulation. Hence, *simultaneous* targeting of multiple possible adaptation methods tackling the same root process may represent the most promising approach in order to prevent adaptation in the first place - very similar, in fact, to finding the right combination of antibiotics for secure and fast eradication of multi-resistant bacteria while preventing further resistances.

The rate-limiting step to (ii) tumor cell *proliferation* and *growth*, in turn, likely includes mitochondrial biogenesis and, thus, ATP generation through mitochondrial OXPHOS in the cancer cell compartment, supported by macronutrient supply from the adjacent glycolytic stromal compartment. Yet again, MYC activation appears to be an important player, as mitochondrial biogenesis is triggered by MYC [148]. In light of the two-compartment model of cancer, it appears absolutely indispensable to target *both* compartments simultaneously and stringently. By conventional chemotherapeutic or radiotherapeutic approaches, this major goal is not achieved: whereas bulk cancer cells may be drastically reduced, CSCs are left to survive and the CAF phenotype is reinforced by great amounts of ROS originating from CTx, which has been suggested to pave the way for relapse and metastasis and to correlate with poor prognosis [34]. Notably, both compartments appear to rely heavily on activated PI3K-AKT-mTOR signaling, which is a major advantage for therapeutic targeting. Therefore, current strategies are focused on small molecular PI3K-pathway inhibitors such as everolimus, which is in use in clinical trials and has already been approved for specific indications in breast cancer patients.

Metformin, another strong mTOR inhibitor and a widely used oral antidiabetic drug, has lately been receiving considerable attention for its cancer-preventive and cancer-therapeutic effects [149–151] and is on its way to being repurposed for preventive and therapeutic use in cancer patients - an example of “drug repositioning” [152]. Metformin is mainly known to impair gluconeogenesis in liver tissue. However, another mechanism of action can be attributed to the inhibition of complex 1 of the respiratory chain [153, 154], which promotes AMPK activation and, ultimately, glycolysis (in fact, one very rare but feared side effect of metformin is lactic acidosis evolving from activated glycolysis combined with impaired gluconeogenesis from lactate). This appears contradictory on first glance. If a tumor was to thrive on Warburg metabolism alone, as has been suggested previously, this mechanism would not be expected to antagonize but instead to drive tumor progression. In the context of two-compartment reverse Warburg tumor metabolism, however, the potential mechanism of action appears obvious: while antagonizing both compartments by strongly and directly inhibiting mTOR, metformin additionally inhibits OXPHOS in cancer cells relying on mitochondria for energy production. In fact, CSCs have been shown to be particularly sensitive towards metformin treatment [30]. Consistent with this, metformin has also been found to downregulate *MYC* mRNA by inducing the expression of ribonuclease *DICER1* and upregulating miRNA-33a in an AMPK-dependent manner [155]. There is evidence, however, that metformin's action in promoting cancer cell death is significantly enhanced when glucose availability is drastically restricted [156]. Metformin itself lowers plasma glucose levels only by about 3 mM (55 mg/dl) in diabetics [157] and does not have any significant glucose-lowering effect in non-diabetics [158]. As patient compliance for strict glucose restriction may not be very high (e.g. adherence to ketogenic or calorie-restricted diets - though probably most natural for humans over the course of millions of years of evolution), an alternative option might be the combined administration of metformin with 2-deoxyglucose, which is taken up in place of glucose but cannot subsequently be used for glycolytic catabolism [159–162]. The enhanced cytotoxicity of this drug combination to cancer cells has been shown for a broad spectrum of cancer models - *in vitro* and *in vivo* [163] - but has, to the best of our knowledge, only been shown experimentally to this date. However, since both drugs have been widely used in clinical diagnostic or therapeutic settings for decades, clinical application of the drug combination should be able to follow promptly. Metformin has further been shown to greatly improve the anti-cancer efficacy of chemotherapeutic drugs commonly used in breast cancer, often allowing reduced dosage regimens [164, 165]. Moreover, combinations with PI3K pathway inhibitors, e.g. rapamycin and everolimus, and with tamoxifen, trastuzumab, and erlotinib have revealed synergistic effects in breast cancer treatment [166–171]. Most likely being of great advantage for the majority of cancers, metformin appears to be particularly well suited for the needs of *BRCA1* germline mutation carriers [149] for the following reasons. (i) BRCA1 is imitated in its role in ACCA inhibition (as metformin activates AMPK, which in turn inhibits ACCA), thereby impairing fatty acid synthesis. (ii) Metformin acts selectively toxic on *TP53*-deficient cells (as is almost always the case in *BRCA1*-associated tumors). (iii) Metformin antagonizes PI3K-AKT-mTOR signaling (e.g. due to additional *PTEN* deficiency) through mTOR inhibition. (iv) Metformin antagonizes *IGF1R* overexpression *via* disruption of androgen signaling, aromatase (and thus E2 generation), and ER expression levels [151, 172, 173], substantially attenuating the double burden on PI3K signaling in *BRCA1* germline mutation carriers.

Another potential treatment option to specifically target mitochondrial biogenesis in CSCs has recently been described by Lamb *et al.* for a long-known class of drugs [174]. Antibiotics known for targeting bacterial protein biosynthesis, including erythromycins and tetracyclines, inhibit mitochondrial biogenesis as a known side effect. Intriguingly, the authors have shown that these antibiotic drugs can be used to specifically eradicate CSCs in 12 different cancer cell lines across eight different tumor types including breast cancer, effectively treating cancer as a “single disease of stemness”. To trace down chemo-resistant CSCs after applied CTx and to target them in their individual vulnerable hotspots, the same group has suggested an innovative method for personalized cancer treatment [175]. First, mitochondrial fluorescent staining is applied to primary tumor or metastatic tissue in order to estimate mitochondrial mass (cells with high mitochondrial mass are also specifically enriched with CSC markers). Then, cells are sorted by flow cytometry in order to isolate mito-high cells (CSCs). Finally, these cells can be tested for sensitivity towards a broad range of chemotherapeutics, mitochondria-targeting antibiotics, metformin or other biguanides, and further drugs. This exciting new treatment concept will likely be a major focus of future research.

## CONCLUSIONS AND OUTLOOK

There have been several attempts to correlate molecular breast cancer subtypes with major metabolic features. For instance, Lloyd *et al.* [176] proposed to classify ER^+^ *versus* ER^−^ tumors into glucose-dependent *versus* glutamine-dependent, respectively. However, results from a recent study by Willmann *et al.* [48] largely contradict this proposal. In this study, the analyzed ER^+^ breast cancer cell line BT-474 showed the lowest glucose consumption and the lowest lactate levels in comparison to all other investigated cell lines, which were ER^−^. Timmerman *et al.* [76] also showed that molecular subtypes of breast cancer cell lines do not neatly separate into glucose or glutamine high and low consumers. It is possible that metabolic studies on cell culture models of breast cancer may be heavily biased due to unphysiological conditions such as altered composition and concentration of the cell medium or lack of inclusion of *in vivo* tumor microenvironment, which excludes an important player in tumor metabolism. Thus, cells that only show moderate glucose consumption in an *in vitro* setting may be ones that - in an *in vivo* setting - substantially rely on macronutrients obtained from adjacent CAFs, rather than on their own glycolytic flux, and may therefore be more adapted to alternative fuel sources besides glucose. On the other hand, cells that show high glucose consumption *in vitro* may either be more autonomous in an *in vivo* setting or may profit from adjacent CAFs' metabolism in addition to their own and subsequently exhibit a more aggressive phenotype *in vivo.* Martinez-Outschoorn *et al.* [10] have attempted to diminish this problem by mimicking the breast tumor microenvironment utilizing an *in vitro* co-culture model of human breast cancer cells and immortalized human fibroblasts. Regarding the potential metabolic heterogeneity between cancer cells existing in immediate adjacency to each other in an *in vitro* as well as an *in vivo* setting, cell sorting *via* FACS prior to fractionized metabolome analyses on separate cell types, or even single-cell metabolome analyses, may be mandatory. Great metabolic heterogeneity, as a possible reflection of the individual adaptability of cancer cells, may further demonstrate the necessity to focus in parallel on (i) joint, central pathways that appear rate-limiting to each tumor independent of individual adaptations (e.g. mitochondrial OXPHOS in cancer cells, glycolysis particularly in CAFs, and PI3K signaling in both), and (ii) individual customized treatment by means of specific enzyme and substrate substitution following individual metabolome analyses or through tumor cell sorting with subsequent drug sensitivity screening in a manner similar to antibiogram screening.

As ROS signaling appears to play a major initiating role in the onset of both CAF and CSC phenotypes, one is prone to wonder why patients of distinct mitochondrial disease, which often leads to high amounts of ROS, do not appear to exhibit excessive tumor incidence rates at a young age already. In fact, taking into account the above findings, innate dysfunctional mitochondria could even be protective to the organism in regard to tumor formation, since highly efficient OXPHOS - possibly a prerequisite for rapid proliferation - is essentially impaired. Mitophagy defects, acquired or innate, may indeed confer a dual protective mechanism as (i) CAFs may be hampered in exporting nutrients resulting from autophagic degradation, and (ii) cancer cells may be hindered in acquiring highly productive, powerful mitochondrial mass. Interestingly, for Parkinson's disease, in which defective mitophagy has been found to play a major pathogenic role, a negative correlation between disease prevalence and the incidence of most cancers has indeed been described (reviewed in [177]).

Alas, deeper understanding of the metabolic mechanisms involved in tumorigenesis - perhaps above all the conditioning of stroma cells towards an oxidation-rich, glycolytic, pro-inflammatory, and pro-tumorigenic state - may serve as a basis not only for further elucidation of oncogenic processes, but potentially also for the illumination of a large number of chronic “non-communicable” diseases, interlinking metabolic syndrome and cardiovascular disease, neurodegenerative and neuro-psychiatric disorders, autoimmune and chronic inflammatory disease [178]. All of these conditions have been implicated in similar aging-related metabolic changes, notably aberrant ROS accumulation, inflammation, mitochondrial dysfunction, autophagy-related changes, and an imbalance between pro-proliferative and pro-apoptotic signaling. Alterations within the corresponding underlying cellular pathways, which are physiologically meant to assist regulatory processes (e.g. wound healing, germ-fighting, tissue regeneration, cell survival strategies, and simple growth), present a major focus of ongoing trans-sectoral research and point towards hereditary factors and environmental modifiers being equally contributive operating forces in driving metabolic variance. Further research focusing on disease-associated metabolomic aberrations in a major effort to finding preventive and therapeutic measures against the diseases of our time - in particular common but devastating malignancies such as breast cancer - is warranted.

## Abbreviations

2HG: 2-hydroxyglutarate
3PG: 3-phosphoglycerate
AA: amino acids
Ac-CoA: acetyl coenzyme A
ACCB: acetyl-CoA-carboxylase-beta
AMPK: AMP-activated protein kinase
ASCT2: ASC amino-acid transporter 2
AMP: adenosine monophosphate
ATP: adenosine triphosphate
αKG: alpha-ketoglutarate
CAF: cancer-associated fibroblast
CAV1: caveolin 1
Cys: cysteine
Cys: Cys-cystine
E2: estradiol
EMT: epithelial-mesenchymal transition
ERα: estrogen receptor alpha
ERBB2: erb-b2 receptor tyrosine kinase 2
ERRα: estrogen-related receptor alpha
F1,6BP: fructose 1,6-bisphosphate
F6P: fructose 6-phosphate
FA oxidation: fatty acid oxidation
FA synthesis: fatty acid synthesis
FH: fumarate hydratase
Fum: fumarate
G6P: glucose 6-phosphate
g*BRCA1*m: germline *BRCA1* mutation
GDH: glutamate dehydrogenase
Glc: glucose
Gln: glutamine
GLS: glutaminase
Glu: glutamate
GLUT: glucose transporter
g*PTEN*m: germline *PTEN* mutation
GSH: glutathione (reduced form)
GSSG: glutathione disulfide (oxidized form)
g*TP53*m: germline *TP53* mutation
HIF1α: hypoxia-inducible factor 1-alpha
HIF2α: hypoxia-inducible factor 2-alpha
HK2: hexokinase 2
IDH1/2: isocitrate-dehydrogenase 1/2
IDH1/2 mut: isocitrate-dehydrogenase 1/2 mutant
IGF1: insulin-like growth factor 1
IGF1R: insulin-like growth factor 1 (IGF-1) receptor
Lac: lactose
LAT1: L-type amino acid transporter 1
LDH: lactate dehydrogenase
Leu: leucine
ME: malic enzyme
MCT1: monocarboxylate transporter 1
MCT4: monocarboxylate transporter 4
mTOR: mechanistic target of rapamycin
NADP^+^/NADPH: nicotinamide adenine dinucleotide, phosphate-(oxidized and reduced form)
NFĸB: nuclear factor-ĸB
NRF2: nuclear factor E2-related factor 2
OAA: oxaloacetate
OXPHOS: oxidative phosphorylation
PDH: pyruvate dehydrogenase
PEP: phosphoenolpyruvate
PFK2: phosphofructokinase 2
PGC1: peroxisome proliferator-activated receptor γ, coactivator 1
PKM2: pyruvate kinase isozyme M2
ROS: reactive oxygen species
SDH: succinate dehydrogenase
SIRT: sirtuins
Succ: succinate
TGFβ: transforming growth factor beta
TCA: tricarboxylic acid cycle
TSC2: tuberous sclerosis complex 2
ULK1: unc-51-like kinase 1
xCT/SLC7A11: solute carrier family 7 (xc- system), member 11, cystine/glutamate transporter
